# Topological one-way fiber of second Chern number

**DOI:** 10.1038/s41467-018-07817-3

**Published:** 2018-12-19

**Authors:** Ling Lu, Haozhe Gao, Zhong Wang

**Affiliations:** 1 Institute of Physics, Chinese Academy of Sciences/Beijing National Laboratory for Condensed Matter Physics, Beijing, 100190 China; 2 Songshan Lake Materials Laboratory, Dongguan, Guangdong 523808 China; 3 University of Chinese Academy of Sciences, Beijing, 100049 China; 4 Institute for Advanced Study, Tsinghua University, Beijing, 100084 China; 5 Collaborative Innovation Center of Quantum Matter, Beijing, 100871 China

## Abstract

One-way waveguides have been discovered as topological edge states in two-dimensional (2D) photonic crystals. Here, we design one-way fiber modes in a 3D magnetic Weyl photonic crystal realizable at microwave frequencies. We first obtain a 3D Chern crystal with a non-zero first Chern number by annihilating the Weyl points through supercell modulation. When the modulation becomes helixes, one-way modes develop along the winding axis, with the number of modes determined by the spatial frequency of the helix. These single-polarization single-mode and multi-mode one-way fibers, having nearly identical group and phase velocities, are topologically-protected by the second Chern number in the 4D parameter space of the 3D wavevectors plus the winding angle of the helix. This work suggests a unique way to utilize high-dimensional topological physics using topological defects.

## Abstract (web summary)

Topological one-way fibers are promising candidates for novel fiber devices. Here, Lu et al. propose that one-way fiber modes are topologically protected by the second Chern number in a four-dimensional parameter space, which develop in a helically-modulated magnetic Weyl photonic crystal.

## Introduction

Topological photonics^1–4^ started with the realization of one-way edge waveguides^5–9^ as the analog of chiral edge states of the two-dimensional (2D) Chern insulator (or the 2D quantum Hall effect [QHE]), where the number and direction of 1D edge modes are determined by the 2D bulk topological invariant: the first Chern number (*C*_1_). Three-dimensional (3D) bands of nonzero *C*_1_ have also been realized in Weyl photonic crystals^10^, opening doors to 3D topological phases for photons^11,12^.

**Fig. 1:**
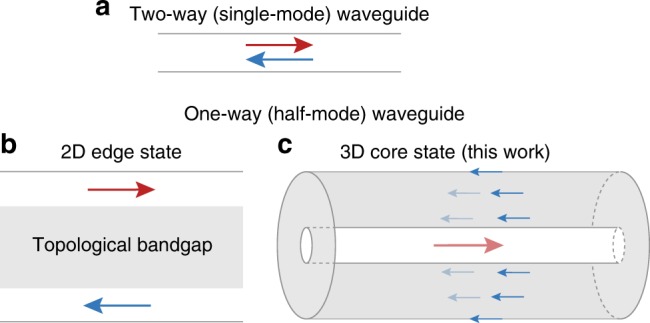
Conceptual sketch of the one-way (half-mode) waveguides in 2D and 3D. **a** Single-mode two-way waveguide. **b** One-way waveguide at the edge of a 2D topological bandgap material, across which the forward and backward modes are spatially separated. **c** One-way fiber at the core of a 3D topological bandgap material. The backward mode is spatially separated, from the forward core mode, at the outer surface of the fiber cladding

Here, we show that, by annihilating a single pair of Weyl points with helix modulations, light can be guided unidirectionally in the core of 3D photonic crystal fibers (Fig. 1), where the number and direction of one-way modes equal the magnitude and sign of the second Chern number (*C*_2_)—the topological invariant of complex vector bundles on 4D manifolds. This novel approach to create the line-defect states in the 3D topological bandgap provides a definitive way to obtain arbitrary mode number (*C*_2_ = −∞ to +∞) in the one-way fibers by varying the helix frequencies. Furthermore, all the modal dispersions have almost identical group and phase velocities, superior for multimode operations. The same phenomena can be realized in other Weyl systems^13–17^ with time-reversal symmetry breaking.

## Results

### Single Weyl dipole

**Fig. 2:**
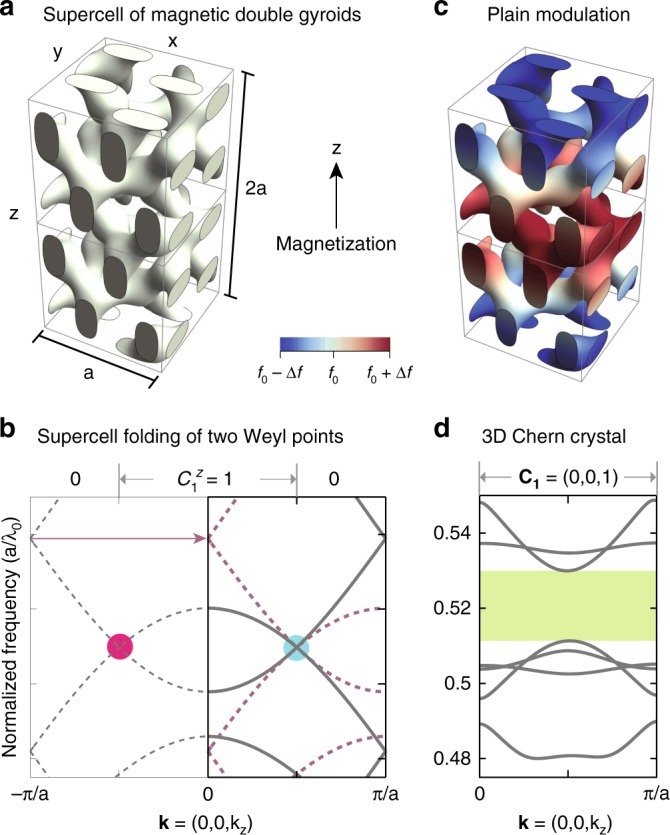
3D Chern crystal from magnetic Weyl crystals. **a** Two cubic unit cells of the DG photonic crystal magnetized along *z*. **b** The band structure of a cubic cell shows two Weyl points, which fold into one 3D Dirac point in the Brillouin zone of the supercell. **c** The DG photonic crystal whose volume fraction (blue–red colored) is periodically modulated along *z*. **d** The band structure of the 3D Chern crystal whose topological gap frequencies are highlighted in green. *λ*_0_ is the vacuum wavelength

Our starting point is a photonic crystal containing two Weyl points^18^, which were found in the double gyroid (DG) made of magnetic materials. The DG is a minimal surface that can be approximated by the iso-surface of a single triply periodic function: *f*(*x*, *y*, *z*) = sin(2*πx*/*a*)sin(4*πy*/*a*)cos(2*πz*/*a*) + sin(2*πy*/*a*)sin(4*πz*/*a*)cos(2*πx*/*a*) + sin(2*πz*/*a*)sin(4*πx*/*a*)cos(2*πy*/*a*). This definition, although having a different form, yields almost identical geometry and band structure to those of the DG defined in ref. ^18^ by two separate trigonometric functions (one for each gyroid). In Fig. 2a, two cubic cells of the DG are shown, where *f*(*x*, *y*, *z*) > *f*_0_ = 0.4 is filled with gyroelectric material of dielectric constant $$\epsilon = \left( {\begin{array}{*{20}{c}} {17} & { - 6i} & 0 \\ { + 6i} & {17} & 0 \\ 0 & 0 & {16} \end{array}} \right)$$ and unity magnetic permeability. The rest of the volume is air. In this structure, there exists only two Weyl points (a single “Weyl dipole”) separated by about half of the Brillouin zone along *z* direction, as plotted in Fig. 2b. This means that an infinitesimal supercell modulation of the crystal (in *z* with a period of 2a) can superimpose the two Weyl points on top of each other to form a 3D Dirac point between four bands^11^ (Fig. 2b), which opens a gap under a finite modulation/coupling strength (Fig. 2d). The fact that a bandgap does not close under small perturbations ensures the robustness of this approach: certain mismatch between the Weyl-point separation and the wavevector of the modulation can be tolerated.

### 3D Chern crystal

We create a double-cell periodic modulation along *z* to annihilate the Weyl points and obtain the 3D Chern crystal, the photonic analog of the 3D Chern insulator (or the 3D QHE)^19–21^. (So far, experimental realization of 3D Chern insulators is only limited to quasi-2D systems^22^). This modulation can be implemented in various system parameters, such as volume fraction, refractive index, magnetization, or structural distortion. In this work, we modulate the volume fraction of the DG by modifying the DG equations as follows: *f*(*x*, *y*, *z*) > *f*_0_ + Δ*f* cos(*πz*/*a*), in which Δ*f* = 0.07. The modulated DG is shown in Fig. 2c and its band structure is plotted in Fig. 2d.

A 3D Chern crystal is characterized by three first Chern numbers ${\bf{C}}_{\bf{1}} = \left( {C_1^x,C_1^y,C_1^z} \right)$ defined on the $\hat x$, $\hat y$, and $\hat z$ momentum planes. For example, $C_1^z$ is defined as

1 $$C_1^z \equiv C_1^z\left( {k_z} \right) = \frac{1}{{2\pi }}{\int} {\kern 1pt} d^2k{\mathrm{Tr}}\left[ {{\cal F}_{xy}} \right].$$

Because the bulk spectrum is gapped, $C_1^z$ cannot change as a function of *k*_*z*_. When there are *N* bulk bands below the bandgap, ${\cal F}_{xy}$ is an *N* × *N* matrix, whose elements are ${\cal F}_{xy}^{\alpha \beta } = \partial _x{\cal A}_y^{\alpha \beta } - \partial _y{\cal A}_x^{\alpha \beta } + i[ {{\cal A}_x,{\cal A}_y} ]^{\alpha \beta }$, in which *α*, *β* = 1, 2, ⋯, *N*. The Berry connection ${\cal A}_i^{\alpha \beta }({\bf{k}}) = - i\left\langle {u^\alpha ({\bf{k}})} \right|{\textstyle{\partial \over {\partial k_i}}}\left| {u^\beta ({\bf{k}})} \right\rangle$, where $\left| {u^{\alpha (\beta )}} \right\rangle$ are the periodic part of the Bloch wavefunctions (see ref. ^1^ for an introduction). Note that the trace of the commutator ${\mathrm{Tr}}[ {{\cal A}_x,{\cal A}_y} ]$ always vanishes for the first Chern class.

The topological invariants of our plainly modulated DG is **C**_**1**_ = (0, 0, 1). This can be understood from the original Weyl photonic crystal where $C_1^z = 1$ for half of its Brillouin zone, as illustrated in Fig. 2b. By folding the Brillouin zone to half of its original size, the Chern numbers in different regions add up.

The 3D Chern crystal is a weak topological phase whose weak topological invariants are defined in a lower dimension, as compared with a strong topological phase with a strong topological invariant. It is theoretically known that a lattice dislocation in a weak topological phase creates a 1D topological defect mode^23^. Unfortunately, in our case, a dislocation induces significant lattice distortion that generates many additional non-topological modes in the bandgap.

Fortunately, we propose and demonstrate below that, for a 3D Chern crystal constructed from Weyl crystals, a new approach is available: a smooth helical modulation generates a one-way mode at the core of the helix. The advantage of the helical-modulation approach, compared with the lattice-dislocation approach, is the intactness of the lattice that prevents the generation of non-topological modes in the bandgap. We outline a physical interpretation as follows, and the rigorous calculations are presented in the Methods section. A supercell modulation couples two Weyl points of opposite chiralities, forming a gapped 3D Dirac point with a mass term that is complex-valued. (A 3D Dirac point consists of two Weyl points of opposite chiralities.) Then a helical modulation amounts to a nonzero winding number for the phase of the Dirac mass around the helical axis. It was indicated, in previous theoretical models, that such a topological perturbation can generate topological defect modes in both 2D^24–26^ and 3D systems^27–30^.

### One-way fiber modes

Now comes the crucial step in our design of topological one-way fibers. Instead of the plain modulation (Fig. 2c), we create a helical modulation by filling the volume satisfying the inequality

2 $$f(x,y,z) \,> \, f_0 + {\mathrm{\Delta }}f{\kern 1pt} {\mathrm{cos}}(\pi z{\mathrm{/}}a + w\theta ).$$

**Fig. 3:**
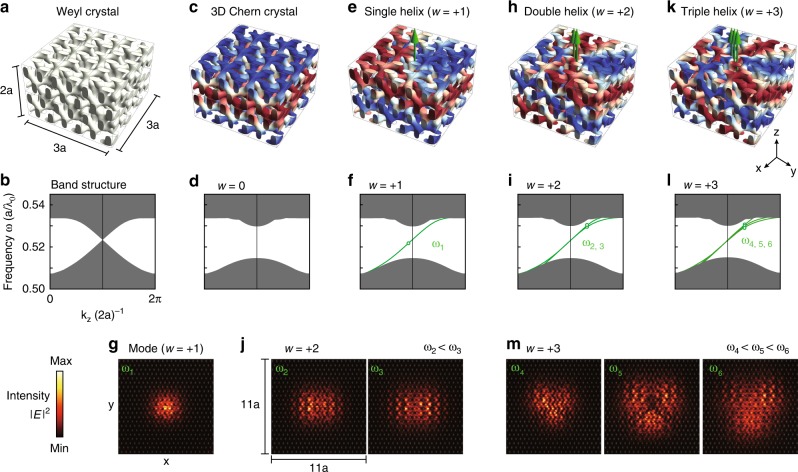
Single and multimode fibers in helically modulated magnetic DGs. **a** The DG structure without modulation is shown in a 3 × 3 × 2 cubic cell and its projected Weyl band structure is shown in **b**. **c** The DG structure of a plain modulation (*w* = 0), and its projected gapped band structure is shown in **d**. **e** The single helix DG structure (*w* = +1), whose helix center supports one one-way fiber mode. The fiber dispersion and mode profile are shown in **f**, **g**. **h** The double helix DG structure (*w* = +2), whose helix center supports two one-way fiber modes. The fiber dispersions and mode profiles are shown in **i**, **j**. **k** The triple helix DG structure (*w* = +3), whose helix center supports three one-way fiber modes. The fiber dispersions and mode profiles are shown in **l**, **m**

The modulation now winds as a function of the angle *θ* [arctan(*x*, *y*)] in the *x* − *y* plane, whose spatial frequency is controlled by the signed integer *w*. The sign and magnitude of *w* determines the direction and number of the one-way modes on the winding axes. This is illustrated in the upper panels of Fig. 3 for *w* = +1, +2, +3, corresponding to single, double, and triple helix one-way fibers.

The band structures of the one-way fibers are shown in Fig. 3f, i, l. They were calculated using MIT Photonic Bands on a 11*a* × 11*a* × 2*a* cubic supercell. The spectra exhibit one-way modes within the bulk bandgap. The fields of the topological fiber modes are localized around the helix cores (Fig. 3g, j, m), and the localization length is minimized at the mid-gap frequencies. In general, the higher-order mode profiles are more extended in the real space. For the multimode fields of *w* = +2, +3, instead of the mid-gap frequencies, we plot the mode profiles close to the band-edge frequencies. Because multimode dispersions are almost degenerate at the middle of the bandgap (Fig. 3i, l), it is difficult to resolve their intrinsic mode patterns from their linear superpositions.

In Fig. 3f, i, l, all one-way-fiber dispersions (green lines) have very similar phase and group velocities. In the multimode cases, their dispersions are almost degenerate at the mid-gap frequencies. This is due to the fact that these defect modes originated from the same Weyl bulk bands, so they all share the same Brillouin-zone location and group velocities as those of the original Weyl cones. This behavior is different from that of the multimode one-way edge waveguides in 2D^9^, where the edge modal dispersions have different phase or group velocities. This can be attributed to the fact that the edge environment, of sharp terminations, is distinct from the environment of the 2D bulk lattice. While, here, there are no sharp interfaces in the 3D one-way fibers. This unique feature, of multiple fiber modes having almost identical dispersions, ensures that multimode signals propagate at the same speed for both energy and phase.

### Time-domain simulations

**Fig. 4:**
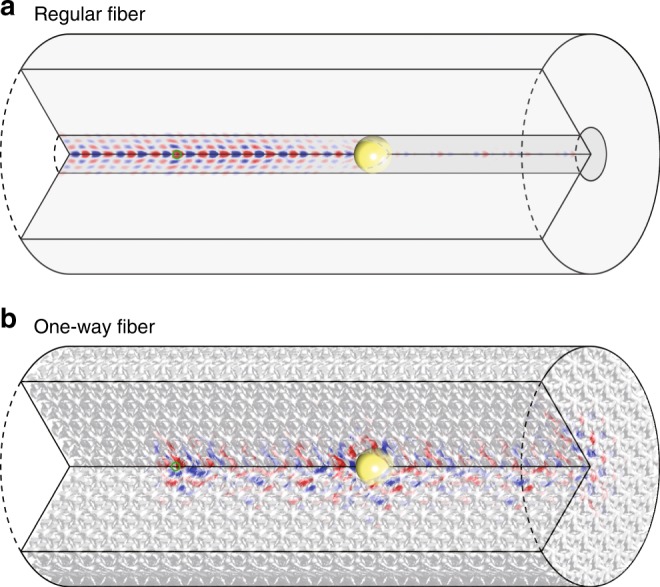
Comparison of a regular fiber and the one-way fiber. **a** A regular two-way fiber mode with strong backscattering off the metallic ball (refer to Supplementary Movie 1). **b** The one-way fiber mode (*w* = +1) has no backscattering (refer to Supplementary Movie 2). The magnetic gyroid photonic-crystal structure is plotted in gray. One-quarter of the fiber volume was removed to expose the electric field of the fiber mode excited by the continuous point source placed at the green circle

To visualize and confirm our prediction made by spectral dispersions, we simulate the wave dynamics of the one-way fiber (*w* = +1) in the real space in Fig. 4. Due to the huge computation domain, the finite-difference time-domain (FDTD) method is adopted for its nice scaling with the computation size. We use the commercial software EastWave^31^ for its capability in handling nonreciprocal materials.

In Fig. 4, we compare the one-way fiber to a regular fiber having a core diameter of 2*a* with a dielectric constant of 16 in air. In both cases, the computation domain is 20*a* × 20*a* × 26.5*a*, in (*x*, *y*, *z*) directions, and the mesh resolution is *a*/30. The perfectly matched layers (PMLs) are used at all six boundary planes. A dipole source polarized in *z* direction was placed at the position (0.1, 0.2, 5.5)*a* to excite the fiber mode. A metallic ball of diameter 1.5*a* is placed at (0, 0, 14.5)*a* to test the robustness of the mode. Obviously, the one-way mode perfectly circumvents the metal sphere without any scattering losses, while the regular fiber mode backscatters. We also note that fiber bends, disrupting the 3D bandgap of cladding, can cause photon loss.

### Second Chern number

It is natural to ask for a topological invariant for the one-way fibers. With the simplest helix modulation of the form of Eq. (2), it is intuitive to guess that *w* is the topological invariant, since the number and direction of the one-way modes match the magnitude and sign of *w*. However, this observation does not work if we consider the modulation of the general form as $f\left( {x,y,z} \right) \,> \,f_0 + \mathop {\sum}\nolimits_w {\kern 1pt} h_w{\kern 1pt} {\mathrm{cos}}\left( {\pi z{\mathrm{/}}a + w\theta } \right)$, where *h*_*w*_ are real-valued constants.

For a lattice dislocation in a 3D Chern crystal, it is known^32^ that the number of chiral modes is given by **C**_1_ · **b**, where the dimensionless Burgers vector (**b**) represents the magnitude and direction of the lattice distortion. However, this approach cannot be applied to our system due to the lack of a unique “Burgers vector” other than that in the simplest case (as of Eq. (2)).

We show that the formal topological invariant of our one-way fibers is the second Chern number (*C*_2_), the strong topological invariant in our system. Note that, far away from the axis of the helix, the Bloch Hamiltonian smoothly varies with *θ* and is a smooth function of the four variables (*k*_*x*_, *k*_*y*_, *k*_*z*_, *θ*). Since (*k*_*x*_, *k*_*y*_, *k*_*z*_, *θ*) span a four-dimensional parameter space with periodic boundary conditions (a 4D torus), the second Chern number^32,33^ can be defined:

3 $$C_2 = \frac{1}{{4\pi ^2}}{\int} {\kern 1pt} d^3kd\theta {\mathrm{Tr}}\left[ {{\cal F}_{xy}{\cal F}_{z\theta } + {\cal F}_{yz}{\cal F}_{x\theta } + {\cal F}_{zx}{\cal F}_{y\theta }} \right].$$

Similar to the definitions in Eq. (1), ${\cal F}_{ij}^{\alpha \beta } = \partial _i{\cal A}_j^{\alpha \beta } - \partial _j{\cal A}_i^{\alpha \beta } + i[ {{\cal A}_i,{\cal A}_j} ]^{\alpha \beta }$, in which *α, β* are the band indices. The non-Abelian Berry potential ${\cal A}_i^{\alpha \beta }({\bf{k}},\theta ) = - i\left\langle {u^\alpha ({\bf{k}},\theta )} \right|\frac{\partial }{{\partial k_i}}\left| {u^\beta ({\bf{k}},\theta )} \right\rangle$, where $\left| {u^{\alpha (\beta )}} \right\rangle$ are the eigenfunctions and *k*_*i*_ runs through *k*_*x*_, *k*_*y*_*, k*_*z*_, *θ*. It is notable that this definition of *C*_2_ involves three variables (*k*_*x*,*y*,*z*_) in the reciprocal space and one variable (*θ*) in the real space, in contrary to the four momentum variables in the 4D QHE^33–40^. Consequently, the Berry curvature ${\cal F}_{i\theta }$ is even while ${\cal F}_{ij}$ is odd under time reversal, where *i* or *j* represents one of *x*, *y*, and *z*. Although in 4D QHE, *C*_2_ can be nonzero without breaking time-reversal symmetry, nonzero *C*_2_ requires time-reversal breaking in our system, which is consistent with the one-way phenomena.

In the Methods section, we carry out the explicit calculations of *C*_2_, which is consistent with our numerical findings in Fig. 3. The topological protection by the second Chern number indicates that the physical origin of the one-way fiber modes is fundamentally different from that of the edge modes of the 2D Chern crystals^5,6^ (2D Chern insulator or 2D QHE), whose topology is captured by the first Chern number. We note that, although in our system both the weak indices (**C**_**1**_) and the strong index (*C*_2_) are nonzero, it is possible to construct a one-way fiber design with zero **C**_**1**_ and nonzero *C*_2_. For example, when the separation between the two Weyl points shrinks to zero (forming a 3D Dirac point), one can apply only angular (*θ*) modulations to obtain a one-way fiber of nonzero *C*_2_ but zero **C**_**1**_.

## Discussion

Experimentally, one-way fibers can be constructed using gyromagnetic materials^6,9,41^ at microwave frequencies. For higher frequencies, there lacks magnetic materials with high Verdet constants and low loss. Nevertheless, we discuss the potential relevant technologies below. Toward optical frequencies, there is progress on magnetic fibers^42,43^ and gyroelectric materials^44,45^. The opt-acoustic coupling^46^ in fibers provides another possibility for breaking time-reversal symmetry. A DG fiber can either be made by drawing a 3D-printed preform or potentially by self-assembly^47,48^ during the drawing process. Three-dimensional direct writing^49^ and interference lithography^50^ can also be adopted. Finally, the chiral modulation can be created by spinning the fiber during drawing, as demonstrated in the chiral fibers^51,52^.

The proposal of one-way fibers enriches the prospects of device applications for the Weyl materials and topological photonics. It also brings a new playground for the realization of higher-dimensional topological physics^53,54^. Topological fibers could also inspire new directions, design principles for novel fibers^55–57^.

## Methods

### Effective 3D Dirac Hamiltonian

In the main text, we have presented our design of the one-way fiber and the results of band-structure calculations. To gain a simple analytical understanding of the one-way modes, we outline below an effective Hamiltonian description, using the low-energy Dirac Hamiltonian. The picture can be summarized as follows. In terms of the effective Dirac Hamiltonian, the modulation corresponds to the presence of a Dirac mass. The helix modulation introduces a topologically nontrivial configuration of the Dirac mass (nonzero winding number of the phase of Dirac mass), which creates topologically one-way defect modes.

In the absence of modulations, the double-gyroid crystal hosts two Weyl points with opposite chirality, either of which is described by a 2 × 2 effective Weyl Hamiltonian. We can combine them as a 4 × 4 block-diagonal Dirac Hamiltonan: *H*_D_ = −*iv*(*σ*_*x*_*∂*_*x*_ + *σ*_*y*_*∂*_*y*_ + *σ*_*z*_*∂*_*z*_)*τ*_*z*_ where *σ*_*i*_,*τ*_*i*_ (*i* = *x*, *y*, *z*) are Pauli matrices, *v* is the group velocity (for simplicity, we take isotropic group velocities with *v* > 0). As we have seen in the main text, a modulation with periodicity 2*a* in the *z* direction gaps out the Weyl points. In the effective Dirac-Hamiltonian description, the frequency gap is due to the Dirac mass terms. There are only two possible Dirac mass terms: *m*_1_*τ*_*x*_ and *m*_2_*τ*_*y*_, both of which anti-commute with the *σ*_*i*_*τ*_*z*_ terms in *H*_D_. It is thus expected that the modulation amounts to the presence of these Dirac mass terms in the low-energy effective Hamiltonian. In general, both *m*_1_ and *m*_2_ can be nonzero, and the mass term can be rewritten as *m*_1_*τ*_*x*_ + *m*_2_*τ*_*y*_ = *mτ*_+_ + m^*^τ_−_, with *τ*_±_ ≡ (*τ*_*x*_ ± *iτ*_*y*_)/2 and *m* ≡ *m*_1_ − *im*_2_. The full effective Hamiltonian, with the effect of modulation included as the Dirac mass, can be written as

4 $$H_{{\mathrm{eff}}} = - iv\left( {\sigma _x\partial _x + \sigma _y\partial _y + \sigma _z\partial _z} \right)\tau _z + m\tau _ + + m^ \ast \tau _ - ,$$

from which we can readily see that a frequency gap 2|*m*| is generated by the Dirac mass terms.

In this effective Hamiltonian, the chirality flipping terms containing *τ*_±_ couple the states near the two Weyl points; therefore, they should come from the modulation. Suppose that the modulation can be modeled in the effective potential $V({\bf{r}}) = V_{\bf{Q}}{\kern 1pt} {\mathrm{exp}}\left( { + i{\bf{Q}} \cdot {\bf{r}}} \right) + V_{\bf{Q}}^\dagger {\mathrm{exp}}\left( { - i{\bf{Q}} \cdot {\bf{r}}} \right) + \cdots$, where **Q** = (0, 0, *π*/*a*) is the wavevector that couples the two Weyl points. We can see that exp(±*i***Q** · ***r***) → *τ*_±_ is valid near the Weyl points, and we have *m* = *V*_**Q**_, in other words, the complex-valued Dirac mass is simply the **Q**-component of the perturbation.

Now, we show that the displacement of modulation (**d**) amounts to the phase change of the Dirac mass (*m*). If we displace the modulation by a distance **d**, then the perturbation becomes *V*(**r** + **d**), which can be expanded as *V*(**r** + **d**) = $V_{\bf{Q}}{\mathrm{exp}}\left[ {i{\bf{Q}} \cdot ({\bf{r}} + {\bf{d}})} \right] + V_{\bf{Q}}^\dagger {\mathrm{exp}}\left[ { - i{\bf{Q}} \cdot ({\bf{r}} + {\bf{d}})} \right] + \cdots$, thus we can see that the displacement causes *V*_**Q**_ → *V*_**Q**_ exp(*i***Q** · **d**), or equivalently, *m* → *m* exp(*i***Q** · **d**). For the helix-shape modulation along the axis *r* = 0, as described in the main text, the displacement **d** is a function of *θ* such that **Q** · **d** = *wθ*. Here, we use the cylindrical coordinates (*x*, *y*, *z*) ≡ (*r*cos*θ*, *r*sin*θ*, *z*). Therefore, we have a nonzero winding of the phase of Dirac mass around the axis, namely, *m*(*θ*) = *m*_0_ exp(*iwθ*), in which *m*_0_ ≡ *m*(*θ* = 0). The overall phase of *m*_0_ can be changed by rotating the coordinate systems around the *r* = 0 axis, thus we are free to take *m*_0_ to be real-valued and positive.

### Analytic solutions of one-way modes

For the effective 3D Dirac Hamiltonian with a nonzero winding of the phase of Dirac mass (with winding number *w*), which is a consequence of the helix perturbation, we shall show that there exist |*w*| topological one-way modes. We can rewrite Eq. (4) in the cylindrical coordinates as

5 $$\begin{array}{l}H_{{\mathrm{eff}}} = \\ \left[ {\begin{array}{*{20}{c}} {vk_z,} & { - ive^{ - i\theta }\left( {\frac{\partial }{{\partial r}} - \frac{i}{r}\frac{\partial }{{\partial \theta }}} \right),} & {m_0e^{iw\theta },} & 0 \\ { - ive^{i\theta }\left( {\frac{\partial }{{\partial r}} + \frac{i}{r}\frac{\partial }{{\partial \theta }}} \right),} & { - vk_z} & {0,} & {m_0e^{iw\theta }} \\ {m_0e^{ - iw\theta },} & {0,} & { - vk_z,} & {ive^{ - i\theta }\left( {\frac{\partial }{{\partial r}} - \frac{i}{r}\frac{\partial }{{\partial \theta }}} \right),} \\ {0,} & {m_0e^{ - iw\theta },} & {ive^{i\theta }\left( {\frac{\partial }{{\partial r}} + \frac{i}{r}\frac{\partial }{{\partial \theta }}} \right)} & {vk_z} \end{array}} \right].\end{array}$$

where we have taken advantage of the translational symmetry in the *z* direction by replacing −*i∂*_*z*_ by *k*_*z*_. For notational simplicity, we shall keep implicit the common factor exp(*ik*_*z*_*z*) in the eigenfunction. For a reason that will become clear shortly, we look for eigenfunctions of the form of *ψ* = [*ψ*_1_, 0, 0, *ψ*_4_]^*T*^. The eigenvalues are *E* = *vk*_*z*_, and the eigenfunctions satisfy

6 $$\begin{array}{l} - ive^{i\theta }\left( {\frac{\partial }{{\partial r}} + \frac{i}{r}\frac{\partial }{{\partial \theta }}} \right)\psi _1 + m_0e^{iw\theta }\psi _4 = 0,\\ m_0e^{ - iw\theta }\psi _1 + ive^{ - i\theta }\left( {\frac{\partial }{{\partial r}} - \frac{i}{r}\frac{\partial }{{\partial \theta }}} \right)\psi _4 = 0.\end{array}$$

It is not difficult to observe that the second equation is equivalent to the first one if we take $\psi _4 = \pm \psi _1^ \ast$. Let us focus on the $\psi _4 = + \psi _1^ \ast$ case first. With this condition, the above two equations are reduced to a single equation

7 $$- ive^{i\theta }\left( {\frac{\partial }{{\partial r}} + \frac{i}{r}\frac{\partial }{{\partial \theta }}} \right)\psi _1 + m_0e^{iw\theta }\psi _1^ \ast = 0$$

For the *w* = +1 case, the common exp(*iθ*) factors can be eliminated; thus, the equation becomes especially simple, and the solution can be found analytically as

8 $$\left| {\psi _{w = + 1}} \right\rangle = \left( {\begin{array}{*{20}{c}} {e^{i\pi /4}} \\ 0 \\ 0 \\ {e^{ - i\pi /4}} \end{array}} \right)e^{ - \frac{{m_0}}{v}r}.$$

For an arbitrary integer *w* ≥ +1, by analysis analogous to ref. ^24^, we can show that there exist *w* localized modes. In fact, we can take the following ansatz for Eq. (7):

9 $$\psi _1^{(l)} = e^{i\pi /4}\left[ {u_le^{il\theta } + v_le^{i(w - 1 - l)\theta }} \right],$$

with an integer parameter *l*, whose acceptable values are to be determined. We first notice that when *l* = (*w* − 1)/2, *e*^*ilθ*^ and *e*^*i*(*w*−1−*l*)*θ*^ are actually equal, and the *v*_*l*_ term is redundant. Let us first focus on the cases *l* ≠ (*w* − 1)/2. The special case *l* = (*w* − 1)/2, with *e*^*ilθ*^ = *e*^*i*(*w*−1−*l*)*θ*^, will be discussed separately later.

According to Eq. (7), the coefficient functions *u*_*l*_,*v*_*l*_ have to satisfy

10 $$\begin{array}{c}v\left( {\frac{\partial }{{\partial r}} - \frac{l}{r}} \right)u_l + m_0v_l = 0\\ v\left( {\frac{\partial }{{\partial r}} - \frac{{w - 1 - l}}{r}} \right)v_l + m_0u_l = 0.\end{array}$$

The asymptotic behaviors of *u*_*l*_, *v*_*l*_ in the *r* → 0 limit can be found as (I) *u*_*l*_ : *r*^*l*^, *v*_*l*_ ~ *r*^*l*+1^ or (II) *u*_*l*_ : *r*^*w*−*l*^, *v*_*l*_ : *r*^*w*−*l*−1^. On the other hand, the asymptotic behaviors in the *r* → ∞ limit are (a) $u_l \to {\mathrm{exp}}\left( { - \frac{{m_0}}{v}r} \right),v_l \to {\mathrm{exp}}\left( { - \frac{{m_0}}{v}r} \right)$ or (b) $u_l \to {\mathrm{exp}}\left( {\frac{{m_0}}{v}r} \right),v_l \to {\mathrm{exp}}\left( {\frac{{m_0}}{v}r} \right)$, only the first of which is normalizable in the *r* → ∞ regime. A normalizable solution must have behavior (a) in the *r* → ∞ limit, which is generally a superposition of (I) and (II) in the *r* → 0 regime. Therefore, the normalizability of the solution in the *r* → 0 limit requires that both (I) and (II) are normalizable, which leads to the constraint

11 $$0 \le l \le w - 1.$$

Thus, we have proved that, leaving out the special case *l* ≠ (*w* − 1)/2 undetermined, there exists one normalizable solution for every integer *l* = 0, 1, 2, ⋯, *w* − 1. However, the solutions with *l* > (*w* − 1)/2 are redundant, because the solutions for *l* and *l*′ with *l* + *l*′ = *w* −1 are actually the same one, as can be appreciated from Eq. (9). Therefore, the total number of solutions with $\psi _4 = + \psi _1^ \ast$ is the number of a nonnegative integer smaller than (*w* − 1)/2, which is [*w*/2] (here, “[⋯]” denotes the floor function, mapping a real number to the largest previous integer), excluding a possible solution with *l* = (*w* − 1)/2.

Now we consider the other choice: $\psi _4 = - \psi _1^ \ast$. By calculations similar to the case $\psi _4 = \psi _1^ \ast$, we can obtain equations similar to Eq. (7), except that the “+” sign before *m*_0_ is replaced by “−”. We adopt the same ansatz as given in Eq. (9), and follows the steps below Eq. (9), solving the case *l* ≠ (*w* − 1)/2 first. It is found that the number of solutions is [*w*/2].

Finally, we study the special case *l* = (*w* − 1)/2 (this case needs consideration only when *w* is odd; for *w* even, this option is automatically absent). Given this value of *l*, the second term in Eq. (9) becomes redundant, thus we can take

12 $$\psi _1^{(l)} = e^{i\pi /4}u_le^{il\theta }.$$

Under the choice $\psi _4 = \pm \psi _1^ \ast$, we obtain the single differential equation

13 $$v\left( {\frac{\partial }{{\partial r}} - \frac{l}{r}} \right)u_l \pm m_0u_l = 0.$$

For the choice “+” of the “±”, Eq. (13) has a single normalizable solution with asymptotic behaviors *u*_*l*_ → *r*^*l*^ in the *r* → 0 limit and $u_l \to {\mathrm{exp}}\left( { - \frac{{m_0}}{v}r} \right)$ in the *r* → ∞ limit. For the choice “−” of the “±”, Eq. (13) leads to $u_l \to {\mathrm{exp}}\left( {\frac{{m_0}}{v}r} \right)$ in the *r* → ∞ limit, which is apparently not normalizable. Therefore, there exists a single normalizable localized mode, in the $\psi _4 = + \psi _1^ \ast$ sector, for the special case *l* = (*w* − 1)/2. We also note that, if we take *m*_0_ < 0 instead of *m*_0_ > 0, the normalizable solution would be present in the $\psi _4 = - \psi _1^ \ast$ sector but absent in the $\psi _4 = + \psi _1^ \ast$ sector, thus the total number of solution is the same.

Let us summarize the above calculations as follows. When *w* is odd, the total number of normalizable solutions is 2[*w*/2] + 1 = *w*; when *w* is even, the total number of normalizable solutions is 2[*w*/2] = *w*. Therefore, the total number of topological modes is always *w*, irrespective of the parity (odd/even) of *w*. Furthermore, our calculation shows that the eigenvalues take the simple form

14 $$E\left( {k_z} \right) = + vk_z,$$

Thus, all these *w* modes propagate along the +*z* direction, with the same velocity *v*.

Finally, let us discuss the one-way modes for the integer *w* < 0. A solution of the form of *ψ* = [*ψ*_1_, 0, 0, *ψ*_4_]^*T*^ does not exist in this case, because the condition given in Eq. (11) can never be satisfied. On the other hand, solutions of the form of *ψ* = [0, *ψ*_2_, *ψ*_3_, 0]^*T*^ can be found. In fact, we can follow the steps above and obtain the equations

15 $$\begin{array}{c} - ive^{ - i\theta }\left( {\frac{\partial }{{\partial r}} - \frac{i}{r}\frac{\partial }{{\partial \theta }}} \right)\psi _2 + m_0e^{iw\theta }\psi _3 = 0,\\ m_0e^{ - iw\theta }\psi _2 + ive^{i\theta }\left( {\frac{\partial }{{\partial r}} + \frac{i}{r}\frac{\partial }{{\partial \theta }}} \right)\psi _3 = 0,\end{array}$$

whose complex conjugations are

16 $$\begin{array}{c} - ive^{i\theta }\left( {\frac{\partial }{{\partial r}} + \frac{i}{r}\frac{\partial }{{\partial \theta }}} \right)\psi _2 + m_0e^{i|w|\theta }\left( { - \psi _3} \right) = 0,\\ m_0e^{ - i|w|\theta }\psi _2 + ive^{ - i\theta }\left( {\frac{\partial }{{\partial r}} - \frac{i}{r}\frac{\partial }{{\partial \theta }}} \right)\left( { - \psi _3} \right) = 0.\end{array}$$

We can see that Eq. (16) is the same as Eq. (6) except that *ψ*_2_ and −*ψ*_3_ take the place of *ψ*_1_ and *ψ*_4_, respectively. Now, our previous analysis for Eq. (6) with *w* ≥ + 1 immediately tells us that the number of one-way modes for *w* < 0 is |*w*|. Because the solutions for *w* < 0 take the form of *ψ* = [0, *ψ*_2_, *ψ*_3_, 0]^*T*^, the dispersion is *E*(*k*_*z*_) = −*vk*_*z*_, thus all the one-way modes propagate in the −*z* direction.

### Calculation of the second Chern number *C*_2_

The effective Hamiltonian Eq. (4) takes the form of

17 $$H_{{\mathrm{eff}}} = \mathop {\sum}\limits_{a = 1}^5 {\kern 1pt} d_a{\mathrm{\Gamma }}^a,$$

where the Dirac matrices Γ^*a*^ = *σ*_*a*_*τ*_*z*_ (*a* = 1, 2, 3), Γ^4^ = *τ*_*x*_, Γ^5^ = *τ*_*y*_, the coefficient functions *d*_*a*_ = *vk*_*a*_ (*a* = 1, 2, 3), *d*_4_ = Re(*m*), *d*_5_ = −Im(*m*). A straightforward calculation of *C*_2_, as in ref. ^33^, leads to

18 $$\begin{array}{c}C_2 = \frac{3}{{8\pi ^2}}{\int} {\kern 1pt} d\theta d^3k{\kern 1pt} \epsilon ^{abcde}\hat d_a\frac{{\partial \hat d_b}}{{\partial k_x}}\frac{{\partial \hat d_c}}{{\partial k_y}}\frac{{\partial \hat d_d}}{{\partial k_z}}\frac{{\partial \hat d_e}}{{\partial \theta }}\\ = \frac{1}{{2\pi }}{\int}_0^{2\pi } {\kern 1pt} d\theta \frac{{d\left[ {{\mathrm{arg}}(m(\theta ))} \right]}}{{d\theta }},\end{array}$$

where $\hat d_a = d_a{\mathrm{/}}\sqrt {\mathop {\sum}\nolimits_{b = 1}^5 d_b^2}$. With the Dirac mass *m* = *m*_0_ exp(*iwθ*), we have

19 $$C_2 = w.$$

For a general modulation that combines several different spatial frequencies, namely, $m(\theta ) = \mathop {\sum}\nolimits_w {\kern 1pt} m_w{\mathrm{exp}}(iw\theta )$, Eq. (18) is not amenable to further simplification in the generic cases. However, we have *C*_2_ = *w*_0_ in the cases that $| {m_{w_0}} | \,> \mathop {\sum}\nolimits_{w \ne w_0} | {m_w} |$, in other words, *C*_2_ is determined by the dominant modulation.

## Supplementary information

Peer Review File

Description of Additional Supplementary Files

Supplementary Movie 1

Supplementary Movie 2

## Data Availability

All relevant data are available from the authors on request.
