# Supplementary material for: Topological one-way fiber of second Chern number
Source: Nat Commun. 2018 Dec 19;9:5384. doi: 10.1038/s41467-018-07817-3 (PMC6300610; doi:10.1038/s41467-018-07817-3)
Supplement: Supplementary file 2 — Description of Additional Supplementary Files [file 41467_2018_7817_MOESM2_ESM.pdf]

Description of Additional Supplementary Files

Supplementary Movie 1
FDTD simulation of the regular two-way fiber mode with a metallic sphere on its path.

Supplementary Movie 2
FDTD simulation of the topological one-way fiber mode with a metallic sphere on its path.
