# Supplementary material for: Topological one-way fiber of second Chern number
Source: Nat Commun. 2018 Dec 19;9:5384. doi: 10.1038/s41467-018-07817-3 (PMC6300610; doi:10.1038/s41467-018-07817-3)
Supplement: Supplementary file 1 — Peer Review File [file 41467_2018_7817_MOESM1_ESM.pdf]

Reviewers' comments:

Reviewer #1 (Remarks to the Author):

In the proposal by Lu and Wang, the authors of the manuscript in question, they show that by using a helical modulation to eliminate Weyl points and open up a three-dimensional Hall effect-type band gap, one-way modes emerge. They propose this as a way of making one-way fibers to be used for technological applications. The scheme is based on a structure similar to that of reference 18 in the text (namely a 3d structure with Weyl points). The structure requires either gyroelectric or gyromagnetic materials to break Lorenz reciprocity. While the physics is very interesting, I don't believe this manuscript is suitable for publication for a number of reasons: [1] I find the proposal by the authors to be extremely far-fetched in practice; [2] there are much simpler ways of making one-way waveguides that actually have the chance to be realized, and [3] from a practical point of view, the modes seem to be highly complex and therefore will be of poor quality at the output.

Currently, optical fibers are fabricated in a draw tower so they can be produced over long lengths and in large qualities. The type of structure proposed here – namely a 3d gyromagnetic or gyroelectric photonic crystal – has never been fabricated (and nothing has come close). I simply cannot imagine that a structure with strong enough Lorenz-breaking could be realized to sustain this effect. To compound this point, the authors use very unrealistic simulation parameters (epsilon=17 on the diagonal of the permittivity tensor). Moreover, the authors go into detail on scattering losses, but what about absorption losses? The current geometry does not preclude these – and they will certainly be strong in optical Lorenz-breaking materials of any kind.

Moreover, I suggest that there are much easier ways of making one-way waveguides. An obvious example is to take a two-dimensional photonic topological insulator and simply use the protected edge states as one-way 'fibers'. This strikes me as a much simpler structure which is easier to realize in practice (given that Lorenz breaking is a key ingredient of this proposal). Furthermore, photonic crystal fibers have already been shown in practice to be capable achieving optical isolation, and therefore potentially one-way guiding – e.g.: [Nature Photonics 5, 549–553 (2011)]. This is a much simpler geometry, which actually can be realized (and has been).

If the authors believe that this is a reasonable proposal for an optical fiber geometry, I suggest that since they are experimentalists they should make the effort to fabricate and realize it. The demonstration of this new fiber would potentially convince me as the referee that my concerns listed above were not warranted.

Reviewer #2 (Remarks to the Author):

I think the paper is technically sound and innovative enough for Nature Communications. Although the ideas behind this paper can be traced to prior work (e.g. PRB 82, 115120 (2010); PRB 92, 241109 (2015)), this paper is nevertheless the first work that introduces the concept of topological defect mode protected by the second Chern number for photonic systems. I basically recommends acceptance and I trust that the authors can give satisfactory answers to a few rather minor issues listed below for their consideration.
1. The results in Fig. 3 are obtained using periodic supercells. Real fibers have finite diameters. What would be the minimum diameter of the fiber that is required to obtain the desired effect?
2. The authors claimed that "Hollow-core one-way fibers can be made by removing the materials on the helix axis, in which the mode con-finement in the air core is ensured by the 3D photonic bandgap.

The extra air volume in the hollow core may generate trivial fiber modes, which can be tuned away."
This paragraph is a bit vague. The authors may need to elaborate a bit more. Do they need to worry about issues such as impedance matching when they "tune away" the usual fiber modes? Also, the authors claimed that all the modes are having nearly identical group and phase velocities. Is that true when they introduce an air core?

3. In the supplemental information, the authors said
"More importantly, one-way fibers completely remove the necessity of fiber isolators – a bulky but essential component in any fiber systems.Lastly, optical force inside a topological one-way fiber is conservative [1], i.e., there is no propelling force for objects inside the hollow-core due to the absence of back-scattered photons. This could be used for optical trapping in fibers." I don't necessarily agree with these advantages claimed by the authors. The topological fibers considered here are way more complex than isolators. The bottom line is that isolators are immediately available, and there is a big gap between the proposal here and any form of practically implementable technology. By the end of the day, both requires a magnetic field and so I really don't see much advantage. In addition, whether an optical field is "conservative" is itself a rather complex issue, and it should be much easier to use the gradient force to achieve trapping than "the absence of back-scattered photons". A gradient force trap can provide stiffness, which cannot be assured the mere absence of back-scattered photons. I think the authors may not want to make these claims.

Reviewers' comments:

Reviewer #1 (Remarks to the Author):

In the proposal by Lu and Wang, the authors of the manuscript in question, they show that by using a helical modulation to eliminate Weyl points and open up a three-dimensional Hall effect-type band gap, one-way modes emerge. They propose this as a way of making one-way fibers to be used for technological applications. The scheme is based on a structure similar to that of reference 18 in the text (namely a 3d structure with Weyl points). The structure requires either gyroelectric or gyromagnetic materials to break Lorenz reciprocity. While the physics is very interesting, I don't believe this manuscript is suitable for publication for a number of reasons: [1] I find the proposal by the authors to be extremely far-fetched in practice; [2] there are much simpler ways of making one-way waveguides that actually have the chance to be realized, and [3] from a practical point of view, the modes seem to be highly complex and therefore will be of poor quality at the output.
We really want to clarify that our theoretical proposal is for microwave experiments, not for optical wavelengths.
In the previous version of our manuscript, our presentation may have caused a misimpression that the work is about new "optical" fibers. In fact, we agree with the reviewer that the current proposal is not for applications in "optical" frequencies. We also thank the reviewer for considering "the physics is very interesting".

In order to clarify this potential misimpression for readers, we have now

A) removed everything throughout the paper containing "optical" or "optical fiber".
B) commented in the "experimental feasibility" section that this proposal is not attainable at the optical frequencies with current materials.
C) noted in the abstract that "They are readily realizable at microwave frequencies with magnetic materials.".

Importantly, our paper contains the following novelties in physics that warranties its publication. We hope the reviewer could have a new view of this paper.

1) We created the first photonic design of the 3D quantum Hall phase by annihilating two Weyl points.
2) We proposed the first material realization of a defect state protected by the second Chern number, without resorting to artificial dimensions.

3) We worked out the first 3D mechanism for topologically-protected one-way photonic state. The previous 2D mechanism was proposed by Haldane that started the field of topological photonics.
4) We provided the first concrete 3D system that can contain arbitrary number of topological one-way states at will. In addition, all the topological modes have identical phase and group velocities, which are very unique properties.

Currently, optical fibers are fabricated in a draw tower so they can be produced over long lengths and in large qualities. The type of structure proposed here – namely a 3d gyromagnetic or gyroelectric photonic crystal – has never been fabricated (and nothing has come close). I simply cannot imagine that a structure with strong enough Lorenz-breaking could be realized to sustain this effect. To compound this point, the authors use very unrealistic simulation parameters (epsilon=17 on the diagonal of the permittivity tensor). Moreover, the authors go into detail on scattering losses, but what about absorption losses? The current geometry does not preclude these – and they will certainly be strong in optical Lorenz-breaking materials of any kind.
Again, we agree with the reviewer and emphasize that the current experimental relevance is for microwave to terahertz frequencies. Regarding the absorption loss, it is determined by the magnetic material loss. We know well from 2D experiments that the propagation length is on the order of 10^2~10^3 periods with the current material choice at tens of GHz.

Moreover, I suggest that there are much easier ways of making one-way waveguides. An obvious example is to take a two-dimensional photonic topological insulator and simply use the protected edge states as one-way 'fibers'. This strikes me as a much simpler structure which is easier to realize in practice (given that Lorenz breaking is a key ingredient of this proposal). Furthermore, photonic crystal fibers have already been shown in practice to be capable achieving optical isolation, and therefore potentially one-way guiding – e.g.: [Nature Photonics 5, 549–553 (2011)]. This is a much simpler geometry, which actually can be realized (and has been).
We thank the reviewer for bring up this recent paper that we have added into our reference list. Indeed, opto-acoustic coupling could potentially be a mechanism for designing one-way fibers in the optical domain in the future.

Compared with the 2D edge state, we proposed a new 3D line-defect state that is protected by a 4D topological invariant, with arbitrary multi-mode creation, and identical

modal phase and group velocities. The current geometry may look complex, nevertheless, it represents a fundamentally new mechanism of generating chiral electromagnetic modes that can inspire simpler designs.

If the authors believe that this is a reasonable proposal for an optical fiber geometry, I suggest that since they are experimentalists they should make the effort to fabricate and realize it. The demonstration of this new fiber would potentially convince me as the referee that my concerns listed above were not warranted.
As we clarified above, we agree with the reviewer that this is not currently a viable proposal for optical frequencies, which we did not claim in the manuscript and now revised the writing so that this will not cause a misimpression.

Instead, this proposal, of enough novel physics, is readily realizable at microwave frequencies. Given the previous experimental experience on 2D magnetic photonic crystals and 3D Weyl crystals (see 3 references below), we have full confidence that this proposal can be implemented in the lab at microwave frequencies quite soon.
Wang, Chong, Joannopoulos, and Soljačić, Nature 461, 772 (2009).
Skirlo, Lu, Igarashi, Yan, Joannopoulos, and Soljačić, Phys. Rev. Lett. 115, 253901 (2015).
Lu, Wang, Ye, Ran, Fu, Joannopoulos, and Soljačić, Science 349, 622 (2015).

**Finally, we added a recent reference on 3D dielectric topological photonic crystals.
A. Slobozhanyuk, S. H. Mousavi, X. Ni, D. Smirnova, Y. S. Kivshar, and A. B. Khanikaev, Nature Photonics (2016).

Reviewer #2 (Remarks to the Author):

I think the paper is technically sound and innovative enough for Nature Communications. Although the ideas behind this paper can be traced to prior work (e.g. PRB 82, 115120 (2010); PRB 92, 241109 (2015)), this paper is nevertheless the first work that introduces the concept of topological defect mode protected by the second Chern number for photonic systems. I basically recommends acceptance and I trust that the authors can give satisfactory answers to a few rather minor issues listed below for their consideration.
We thank the reviewer for his/her positive view of our work and the recommendation of publication in Nature Communications.

1. The results in Fig. 3 are obtained using periodic supercells. Real fibers have finite diameters. What would be the minimum diameter of the fiber that is required to obtain the desired effect?
The minimum fiber diameter should be sufficiently larger than the spatial profiles of the fiber modes in the core. In general, the mode profiles enlarge as the mode number increases, so the minimum diameter increases with the winding number. Also, if we can find a better design with larger bandgaps, the minimal diameter can shrink. For our current design, we find 15~20 super cells in diameter very safe for experimental realization of mode number of 1, 2, 3 at microwave frequencies. For the single mode case, only 10~15 super cells in diameter should be more than enough. We have commented on this aspect in the revised paper.

2. The authors claimed that "Hollow-core one-way fibers can be made by removing the materials on the helix axis, in which the mode con-finement in the air core is ensured by the 3D photonic bandgap. The extra air volume in the hollow core may generate trivial fiber modes, which can be tuned away." This paragraph is a bit vague. The authors may need to elaborate a bit more. Do they need to worry about issues such as impedance matching when they "tune away" the usual fiber modes? Also, the authors claimed that all the modes are having nearly identical group and phase velocities. Is that true when they introduce an air core?
We thank review for raising this question. In a second thought, we think that tuning away of trivial fiber modes are not practically obvious and may not be even possible when the air core is large. Also, the reviewer is right. After the introduction of air core, the modes will not have overlapping dispersions any more due to the abrupt interfaces.

Consequently, we removed the discussion on the hollow core case in the revised paper.

3. In the supplemental information, the authors said
"More importantly, one-way fibers completely remove the necessity of fiber isolators – a bulky but essential component in any fiber systems.Lastly, optical force inside a topological one-way fiber is conservative [1], i.e., there is no propelling force for objects inside the hollow-core due to the absence of back-scattered photons. This could be used for optical trapping in fibers." I don't necessarily agree with these advantages claimed by the authors. The topological fibers considered here are way more complex than isolators. The bottom line is that isolators are immediately available, and there is a big gap between the proposal here and any form of practically implementable technology. By the end of the day, both requires a magnetic field and so I really don't see much advantage. In addition, whether an optical field is "conservative" is itself a rather complex issue, and it should be much easier to use the gradient force to achieve trapping than "the absence of
back-scattered photons". A gradient force trap can provide stiffness, which cannot be assured the mere absence of back-scattered photons. I think the authors may not want to make these claims.
We thank and agree with the reviewer. We removed away such claims in the supplementary.

Reviewers' comments:

Reviewer #2 (Remarks to the Author):

I read the reply and the revised manuscript. My comments are satisfactorily and fully addressed. I have no further questions.

This paper is technically correct (after the authors dealt with several rather minor issues raised in the original report, including the fact that they are not talking about optical fibers) and innovative. It is the first time that the second Chern number, as a topological invariant, has a "material realization". I agree with the first referee that the proposed fiber structure would be difficult to make (actually even in microwave or THz) and there are probably easier ways to obtain one-way modes. So, it is a blue-sky idea that is mathematically beautiful, probably somewhat impractical but I enjoy reading this paper, and I learn something new from it, and I maintain my original recommendation of acceptance.

Reviewer #3 (Remarks to the Author):

In this work, L. Lu and Z. Wang make a theoretical proposal for a photonic analog of a 3D quantum Hall phase with a non-zero Chern number, using a supercell modulation. In addition, they show that when the modulation becomes a helix, topologically protected edge modes arise along the helix winding axis. The author suggest that this phenomenon may be used to create unidirectional microwave waveguides that are topologically protected against scattering losses.

I find one big issue with the paper: the physics are interesting, but are only partially explored. At the same time, the authors try to sell their proposal as being practically useful at microwave frequencies, but do not demonstrate any real practical advantage over previous 2D designs. Yet, the work is very innovative and interesting, and the paper has the potential to be published in Nature Communications if correctly revised. I give more detailed comments and a possible revision route below.

The main drawback of the work is the very high complexity of the design, combined with the fact that many of the new features claimed require such a complexity. Not only one has to build a double-gyroid photonic crystal out of magnetic material, but the geometry has to be modulated in a helical fashion along the direction of the waveguide. Several interesting new features, like having multimode operation with identical dispersion, come at the cost of increasing the helical period (!), and consequently the design complexity. I agree with the authors that the system can be built, however it is clear that the functionality of a one-way waveguide can already be achieved via much simpler 2D (Chern) topological edge modes, which have already been demonstrated at microwaves. For these reasons, the application potential of the proposal is quite limited, even at microwaves. I would encourage the authors to acknowledge this in a revised version, and tone down the claims that the design is superior in practice.

While the application potential is not, in my opinion, a strength of this work, I think the physics involved are very interesting and should become the only focus of the paper. In particular, the authors should at least fully demonstrate their basic claims and provide a deeper study of the topological wave propagation in such waveguides. I have a few points in mind, all related to the robustness to backscattering:

1) The picture of Fig. 1 showing the functionality of a topological “fiber” and its robustness to defects is never directly demonstrated in the paper. The manuscript would be complete if the authors can come back to their initial picture and perform some kind of 3D scattering simulation in a two-port

(finite in all directions) fiber containing a defect. Right now it looks like the claim of Figure 1 is only supported by very abstract, theoretical arguments.

2) It is not clear to me that the topological mode is protected against disorder in the helical modulation, in particular fluctuations of the periodicity and of the location of the winding axis. Again, full-wave scattering simulations could clear all these doubts.

3) To be useful as a fiber, the waveguide should be resilient to bends. The paper does not study to what extent the waveguide can be bent. I imagine that when the radius of curvature becomes not so large compared to the winding period, problems start to occur.

Reviewers' comments:Reviewer #2 (Remarks to the Author):I read the reply and the revised manuscript. My comments are satisfactorily and fully addressed. I have no further questions.

This paper is technically correct (after the authors dealt with several rather minor issues raised in the original report, including the fact that they are not talking about optical fibers) and innovative. It is the first time that the second Chern number, as a topological invariant, has a "material realization". I agree with the first referee that the proposed fiber structure would be difficult to make (actually even in microwave or THz) and there are probably easier ways to obtain one-way modes. So, it is a blue-sky idea that is mathematically beautiful, probably somewhat impractical but I enjoy reading this paper, and I learn something new from it, and I maintain my original recommendation of acceptance.

We thank the reviewer for his/her open attitude on this theoretical proposal and the recommendation for acceptance.

Reviewer #3 (Remarks to the Author):

In this work, L. Lu and Z. Wang make a theoretical proposal for a photonic analog of a 3D quantum Hall phase with a non-zero Chern number, using a supercell modulation. In addition, they show that when the modulation becomes a helix, topologically protected edge modes arise along the helix winding axis. The author suggest that this phenomenon may be used to create unidirectional microwave waveguides that are topologically protected against scattering losses.

I find one big issue with the paper: the physics are interesting, but are only partially explored. At the same time, the authors try to sell their proposal as being practically useful at microwave frequencies, but do not demonstrate any real practical advantage over previous 2D designs. Yet, the work is very innovative and interesting, and the paper has the potential to be published in Nature Communications if correctly revised. I give more detailed comments and a possible revision route below.

The main drawback of the work is the very high complexity of the design, combined with the fact that many of the new features claimed require such a complexity. Not only one has to build a double-gyroid photonic crystal out of magnetic material, but the geometry has to be modulated in a helical fashion along the direction of the waveguide. Several interesting new features, like having multimode operation with identical dispersion, come at the cost of increasing the helical period (!), and consequently the design complexity. I agree with the authors that the system can be built, however it is clear that the functionality of a one-way waveguide can already be achieved via much simpler 2D (Chern) topological edge modes, which have already been demonstrated at microwaves. For these reasons, the application potential of the proposal is quite limited, even at microwaves. I would encourage the authors to acknowledge this in a revised version, and tone down the claims that the design is superior in practice.

We thank and fully agree with the reviewer for his/her comments. It is not a technological winner due to the current complexity of design, though it provides an alternative and interesting approach. We did revise the relevant parts in the paper to reflect this view.

While the application potential is not, in my opinion, a strength of this work, I think the physics involved are very interesting and should become the only focus of the paper. In particular, the authors should at least fully demonstrate their basic claims and provide a deeper study of the topological wave propagation in such waveguides. I have a few points in mind, all related to the robustness to backscattering:

1) The picture of Fig. 1 showing the functionality of a topological “fiber” and its robustness to defects is never directly demonstrated in the paper. The manuscript would be complete if the authors can come back to their initial picture and perform some kind of 3D scattering simulation in a two-port (finite in all directions) fiber containing a defect. Right now it looks like the claim of Figure 1 is only supported by very abstract, theoretical arguments.

We fully agree with the reviewer for his/her request for a time-domain full-wave simulation, which we started two years ago but met huge difficulties in both software and hardware. In short, the system is too large for frequency-domain solvers in terms of memory capacity and computation power. On the other hand, there are no available time-domain solvers (MEEP nor Luminal FDTD) which deals with gyromagnetic dispersions correctly and reliably. Finally, we worked with a software company (Eastwave) to solve it. The software itself cost us 200K RMB.

Now, we finally pulled this off by showing the following figures in the paper and the corresponding movies are also attached with the paper. The student (Haozhe Gao) who did the FDTD simulations and illustrations has been added to the author list.

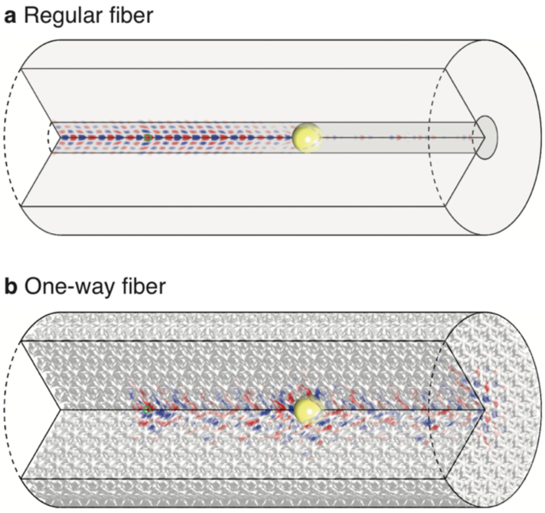

FIG. 4. **Comparison of a regular fiber and the one-way fiber.** a) A regular (two-way) fiber mode with strong back-scattering off the metallic ball. b) The topological one-way fiber mode have no back-scattering. The magnetic gyroid photonic-crystal structure is plotted in gray. One-quarter of the fiber volume was removed to expose the electric field of the fiber mode excited by the continuous point source placed at the green circle.

2) It is not clear to me that the topological mode is protected against disorder in the helical modulation, in particular fluctuations of the periodicity and of the location of the winding axis. Again, full-wave scattering simulations could clear all these doubts.

Indeed, the full-wave simulations we now provide shows robust results. Conceptually, the robustness of the one-way fiber mode is similar to the 2D edge case: disorder on the lattices (without closing the spectral gap) or on the location of the edge *do not* change the one-way behavior.

3) To be useful as a fiber, the waveguide should be resilient to bends. The paper does not study to what extent the waveguide can be bent. I imagine that when the radius of curvature becomes not so large compared to the winding period, problems start to occur.

Indeed, all fibers suffer from bending loss including the one-way fiber.
To be clear, we now add the following sentence in the paper: *We also note that fiber bends, disrupting the 3D bandgap of cladding, can cause photon loss.*

========================

The main changes are:

1) We made a new figure (Fig. 4) showing the simulation results. We also attached the two time-domain movies comparing one-way and regular fibers.
2) We redrew Fig. 1 to be clearer on our novelty in physics, instead of technology.
3) We removed discussions on technological implications.
4) The student (Haozhe Gao) who did the FDTD simulations and illustrations has been added to the author list.

REVIEWERS' COMMENTS:

Reviewer #3 (Remarks to the Author):

I think the authors substantially improved the paper by performing additional simulations to properly support their claims, and refocused their study on the physics. I can therefore recommend publication without reservations.
